# Formulation and Characterization of Potential Antifungal Oleogel with Essential Oil of Thyme

**DOI:** 10.1155/2018/9431819

**Published:** 2018-04-19

**Authors:** Giedre Kasparaviciene, Zenona Kalveniene, Alvydas Pavilonis, Ruta Marksiene, Jurgita Dauksiene, Jurga Bernatoniene

**Affiliations:** ^1^Department of Drug Technology and Social Pharmacy, Lithuanian University of Health Sciences, Sukileliu Pr. 13, LT-50161 Kaunas, Lithuania; ^2^Institute of Pharmaceutical Technologies, Lithuanian University of Health Sciences, Sukileliu Pr. 13, LT-50161 Kaunas, Lithuania; ^3^Institute of Microbiology and Virology, Lithuanian University of Health Sciences, Eiveniu Str. 4, LT-50161 Kaunas, Lithuania; ^4^Department of Analytical and Toxicological Chemistry, Lithuanian University of Health Sciences, Sukileliu Pr. 13, LT-50161 Kaunas, Lithuania

## Abstract

The aim of this research was to formulate oleogel with thyme essential oil with potential antimicrobial activity, design optimal formulation, and evaluate the influence of ingredients on texture parameters of preparation. Central composite design was applied to statistical optimization of colloidal silica and paraffin oil mixture for the modeling of oleogel delivery system. The influence of designed formulations on response variables (texture parameters), firmness, cohesiveness, consistency, and index of viscosity, was evaluated. Quality of essential oil of thyme was assessed by determinate concentration of thymol and carvacrol using gas chromatography with flame ionization detection (GC-FID). Microbiological tests have shown that oleogel with thyme essential oil affects* Candida albicans* microorganism when thyme essential oil's concentration is 0,05% in oleogel mixture.

## 1. Introduction

Complementary and alternative medicines are used by 60–80% of developing countries because they are one of the most prevalent sources of medicine worldwide [1–4]. Essential oils are also one of the most popular natural products, with one of their main applications being their use in dermatology [5–7]. An overview of the research articles about effectiveness of essential oils in topical application states its use for the treatment of infections caused by bacteria, fungi, or viruses in the most cases [8]. Natural topical preparations containing thyme essential oil could be a valuable alternative to antifungal and antibacterial drugs.

*Thymus vulgaris* L. (Lamiaceae) is an aromatic and medicinal plant containing essential oil and is widely used all over the world for its expectorant, antibroncholitic, antispasmodic, and other properties. Antimicrobial properties of plant essential oils were revealed a long time ago, but researches are expanded and complements acknowledged findings. Recent articles present the beneficial properties in nature as biofungicide: thyme essential oil is active against* Mycosphaerella graminicola*—the pathogen today causes one of the most important diseases of wheat [9]. Activity against microorganisms is widely published: against* Staphylococcus aureus*,* Pseudomonas aeruginosa*,* Salmonella typhimurium*,* Escherichia coli*,* Klebsiella pneumoniae*,* Enterococcus faecalis*, and* Candida albicans* [10];* Staphylococcus epidermidis*,* Streptococcus *sp., and* Pantoea *sp. [11]; and* Bacillus *spp.*, Shigella *spp.*, Trichophyton *spp. (nail and scalp isolated),* Microsporum *spp.*, Aspergillus niger, *and* Rhodotorula rubra *[12]. The essential oils are natural, nontoxic, nonpollutive, and biodegradable compounds with broad spectrum of antimicrobial activity, low risk of side effects after their use, and low risk of resistance development by microorganisms [13]. In this investigation, the main goal was focused on activity against* Candida albicans. *Yeasts may act as opportunistic pathogens and can cause candidiasis at several different anatomical sites [14]. Essential oils demonstrating noteworthy activity against this organism are quite extensively investigated and most oils used in dermatology have been tested against this pathogen [8].

A typical essential oil may contain 20 to 80 phytochemicals [15]. Thyme has numerous chemotypes named according to the major compound, for example, thymol, carvacrol, terpineol, and linalool. Thyme essential oil consisted primarily of thymol (38.1%), p-cymene (29.1%), g-terpinene (5.2%), linalool (3.7%), and carvacrol (2.3%) [16]. It has been demonstrated that the biological effects of* Thymus vulgaris* are mainly due to the presence of phenolic compounds, especially thymol and carvacrol [17]. Thymol content in thyme essential oil is much higher than carvacrol content. This compound shows 30 times higher antiseptic effects and four times lower toxicity than phenol [18, 19].

Thyme essential oil is a clear yellow or very dark reddish-brown liquid with a pungent smell of thyme easily mixed with liquid paraffin and vegetable oils. In view of these properties, the form of oleogel was formulated. The majority of oleogel applications reported in the pharmaceutical area were related to transdermal systems, topical bases, and preparations intended for percutaneous absorption [20]. Lipophilic gels are popular in the pharmaceutical industry for simple production technology, long-term stability, and new wide range of gelators. In comparison with emulsions, oleogels have better viscosity and spreadability; the high lipid content of the products causes an optimal reduction of any skin roughness, beneficial for skin protection purposes [21].

Anhydrous colloidal silica is suitable for the formulation of oleogel with desired texture and chemical and physical stability. The clear gel is formed when the material is characterized by a similar refractory index as Aerosil (1.48). Incorporation of the silica into oil leads to an increase in viscosity, which is brought about by hydrogen bonding between the silica particles: 5–10 percent silica imparts a paste-like consistency on a range of oils [21]. Liquid paraffin and olive oil were chosen for the oleogel formulation with the goal of evaluating the influence of the oil origin on the texture and stability. The composition of oleogel was optimized with response surface central composite design in order to get desirable textural properties.

## 2. Materials and Methods

### 2.1. Materials

Oleogel was produced from essential oil of thyme, obtained from Sigma-Aldrich Chemie GmbH (Germany), olive oil, obtained from Henry Lamotte GmbH (Germany), and colloidal anhydrous silica Aerosil 200, obtained from Degussa AG (Germany). For identification of active compounds in oleogels thymol and carvacrol were used, which were obtained from Carl Roth GmbH & Co. Kg (Germany). For HPLC analysis, acetonitrile was used, which was obtained from Chromasolv (USA). Antifungal activity of oleogels was performed on Mueller-Hinton broth II agar, obtained from BBL (USA), and culture* Candida albicans* ATCC 60193, obtained from Becton Dickinson and Company (USA).

### 2.2. Preparation of Oleogel

The gels were prepared by stirring (150 RPM) Aerosil with paraffin and olive oils for 5 min to obtain a homogenous transparent mixture. Essential oil of thyme was added to the oleogel as active substance with antimicrobial activity at a concentration of 0.1% w/w.

### 2.3. Gas Chromatography with Flame Ionization Detection

GC-FID analyses were carried out on gas chromatograph GC-2010 Plus (Shimadzu Corporation, USA). Compounds were separated on Crossbond™ 5% diphenyl/95% dimethyl polysiloxane Rxi®-5 ms, 30 m × 0,25 mmi.d., df 0,25 *μ*m (Restek, USA) capillary column. The injection volume was 1 *μ*l, with a split ratio of 1 : 100. Ultra-high-purity helium was used as a carrier gas at a constant column flow rate of 1,29 ml/min. The injector and detector temperatures were set at 290 and 310°C, respectively. Samples were diluted individually with methanol before GC analysis. The identification of main compounds (thymol and carvacrol) was performed by comparing their retention times of the chromatographic peaks to the reference standards. The main compounds (thymol and carvacrol) were quantified using GC-FID chromatographic data and equations of linear regressions (*R*^2^ ≥ 0.9999) of calibration graphs (area versus concentration of standard). The initial temperature of column was set as 70°C, at which it was held for 2 min. The temperature of the column was programmed from 70°C to 250 at a rate of 20°C/min.

### 2.4. Antimicrobial Activity

The minimum inhibitory concentration (MIC) of the oleogels with essential thyme oil was determined using* Candida albicans* ATCC 60193 culture. Determination was performed by dilution method on plates of solid medium (Mueller-Hinton broth II). Standard fungus culture was cultivated for 20–24 hours at 35–37°C temperature on Mueller-Hinton agar. The suspension is made from the harvested culture in physiological sodium chloride (0.9%) solution, standardized with* McFarland* standard indicator, which measures the turbidity of the suspension in the test tube. Microbial suspensions are standardized when the indicator shows a value of 0.5 (this means that 1 ml of the microbial suspension contains 1.5 × 10^8^ microbial cells). 1 ml of the oleogel was transferred with the sterile pipette into a sterile graduated cylinder, which is mixed with 10 ml of molten rigid Mueller-Hinton medium and transferred to a Petri dish. Concentration of essential oil of thyme ranged from 0.01 to 0.3%. The antifungal effect of investigated gels on* Candida albicans* culture was evaluated after 24 h and compared with control (gel without essential oil).

### 2.5. Experimental Design

The preliminary formulation of the oleogel was prepared for the suitable lower 5% and upper 8.5% limits of colloidal silica concentration for the gelation of oils. The experimental design is conducted by using Design-Expert® 6 (version 6.0.8, Stat-Easy Inc., Minneapolis, USA). A set of mixtures was formed using response surface design central composite criterion. The experimental mixture design was applied to study the effect of two-component system—colloidal silica—on the response variables: firmness, cohesiveness, consistency, and index of viscosity. The set of mixtures is shown in Table 1.

### 2.6. The Texture Analysis

**Figure 1:**
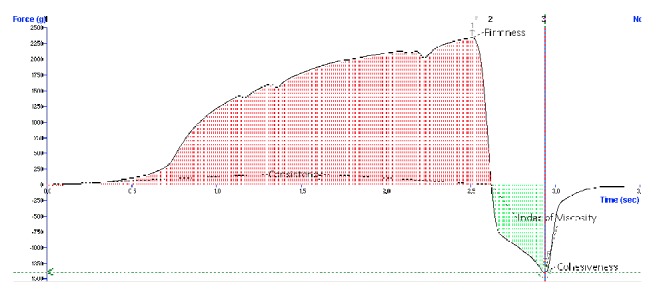
Typical graph of oleogel texture determination using back extrusion test.

The texture profile analysis of the formulated oleogels was conducted using a TA.XT.plus (Stable Micro Systems Ltd., Godalming, Surrey, UK) texture analyzer. Back extrusion test was performed. The back extrusion rig (A/BE) is comprised of a sample container that is centrally located beneath a disc plunger. The disc plunger performs a compression test that extrudes the product up and around the edge of the disc. This test measures the consistency of viscous food products and semisolid pharmaceutical or cosmetic products. When a trigger force of 5 g has been achieved, the disc plunger begins to deform the sample to a specified distance (5 mm) after which the probe returns to its starting position. The maximum force represented as a peak on the graph measures firmness; the higher the value, the firmer the sample (Figure 1). The area under the positive part of the graph indicates sample consistency (work done to hardness 1); the higher the value, the thicker and the higher the consistency of the sample. As the probe returns to its starting position, the initial lifting of the weight of the sample on the upper surface of the disc produces the negative part of the graph resulting from back extrusion. This gives an indication of the cohesiveness and resistance of the sample to separate (flow off) from the disc. The maximum negative force on the graph indicates sample adhesive force; the more negative the value is, the more “sticky” the sample is. The area under the negative part of the graph is known as the adhesiveness (energy required to break probe sample contact) and can give an indication of the cohesive forces of the molecules within the sample. The higher the value, the more energy required to break the probe sample contact as the probe withdraws from sample. All tests were conducted at room temperature (25 ± 2°C) and repeated three times.

### 2.7. Statistical Analysis

The results are presented as mean ± standard deviation. Statistical analysis was performed using Student's *t*-test. A value of *p* < 0.05 was taken as the level of significance.

## 3. Results

### 3.1. Thymol and Carvacrol Amount Determination by Gas Chromatography

Gas chromatography technique provides the industry and science with simplicity, speed, and efficiency for the characterization and quantification of components of essential oils. The instrument most commonly used for identification of the components is a mass spectrometer (MS), while for quantification of the organic compounds it is a flame ionization detector (FID) [22].

**Figure 2:**
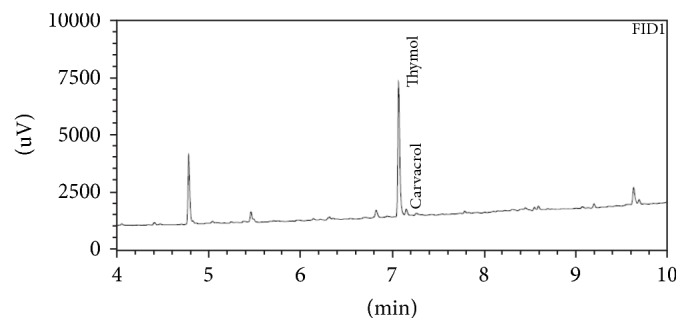
GC-FID chromatogram of oleogel containing* Thymus vulgaris* essential oil.

The quantitative determination of the two main components of the thyme by GC-FID method was applied for the oil and for the oleogels.  Figure 2 demonstrates that thymol and carvacrol peaks were well resolved from each other and displayed excellent peak symmetry and separation efficiency. The concentrations of thymol (9.61 mg/mL) and carvacrol (0.07 mg/mL) were determined in the essential oil. Quantitative analysis of oleogel containing thyme essential oil showed that thymol concentration decreased by 30% and carvacrol concentration decreased by 10% from the initial amount.

### 3.2. Optimization and Texture Evaluation of Oleogels

**Figure 3:**
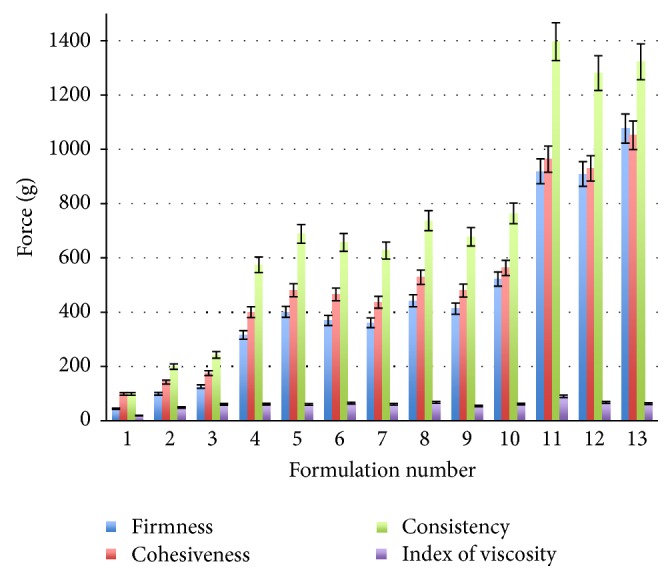
Texture parameters, firmness, cohesiveness, consistency, and index of viscosity, of 13 oleogels formulations.

The experimental design conducted the set of component mixtures for the oleogels with 0.1% essential oil of thyme formulations (Table 1). Evaluation of mechanical properties by texture analyzer was performed as response. Back extrusion test determines firmness, cohesiveness, and consistency and shows what force (g) is achieved. All parameters of the oleogels increased linearly with the increase in the gelator, colloidal silica concentration (Figure 3). Formulations numbers 1, 2, and 3 contained lowest amount of gelator; accordingly, the force used for the determination of texture parameters was up to 250 g. Another group of formulations contained 6.75% of colloidal silica; the texture parameters were of medium values; used force was up to 800 g. Formulations with the highest amount of gelator needed higher force (up to 1400 g) to determinate firmness, cohesiveness, and consistency. Mechanical properties of oleogel depend on the gelator concentration: firmness, cohesiveness, and consistency increased with the increase in the colloidal silica amount. Index of viscosity in all determinations varied from 19.12 ± 0.6 to 89.99 ± 4.5 g and did not show high correlation with amount of oleogel components.

Goals of tested parameters were determinate for the design of optimal formulation: maximum cohesiveness, firmness, consistency, and medium index of viscosity. Oleogel for topical application has to be with good adhesive properties for better permeation to the skin.* Design-Expert*®* 6* program calculated the optimal composition of the oleogel mixture, containing thyme essential oil 0.1%, colloidal silica 8.50%, paraffin oil 25.01%, and olive oil 66.39%. The lack of fit (*F* value < 0.0001) implies that the model is significant and the optimum mixture was suggested with the highest desirability of 0.810.

Optimal oleogel formulation of stated composition was produced and evaluated. It was clear light yellow colour gel with smell of thyme. pH value of oleogel matched human physiological pH value and varied from 6,29 to 6,48. Texture parameters were determined and listed in Table 2.

The relationship between the texture parameters and amounts of oleogel components, colloidal silica, and paraffin oil was evaluated as well. Correlation analysis between main components of oleogel and texture parameters was performed using Spearman's rank coefficient. Colloidal silica as the most important component due to the gelation ability showed high influence on the texture parameters. Correlation coefficients between the amount of Aerosil and cohesiveness, consistency, firmness, and index of viscosity were *R* = 0.978, *R* = 0.977, *R* = 0.966, and *R* = 0.726, respectively.

### 3.3. Antimicrobial Test

The antimicrobial assay of the oleogels with essential oil of thyme was performed against* Candida albicans. *The minimum inhibitory concentration was determined after incubating the oleogels with different concentrations of thymol in Petri dishes. The results are listed in Table 3. The results suggest that the oleogel may be tried as matrices for drug delivery or can be alternative preparation for the chemical drugs.

MIC of thyme oil of 0.25% was observed on the growth of* Candida albicans *strain on solid medium and colonial morphology differed from control, especially in size, and the smallest colonies were detected in the presence of thyme oil with concentration of 0.125% [13]. Concentration of thymol in the essential oil varies from 20% to 54% in all species of thyme and* Thymus vulgaris* as well. Plenty of results of biological activity dependence on concentrations of active substances are published. According to the results of quantitative analysis of oleogel, which demonstrated decreasing concentration of active ingredients, and antimicrobial test results of this study, it was decided to formulate oleogels with 0.1% of essential oil of thyme.

## 4. Discussion

Various studies investigated compositions and biological activity of essential oils and presented valuable results, which encourages the development of products with them. The isolation yield and chemical composition of the essential oils depend on many factors; therefore biological activity can vary. Thyme essential oil can contain 36–55% of thymol. Thyme essential oil has several chemotypes named according to the major compound: thymol, carvacrol, terpineol, and linalool. The concentration of thymol in Romanian thyme essential oil was 47.59% and its activity against* Staphylococcus aureus*,* Salmonella typhimurium*,* Pseudomonas aeruginosa*,* Escherichia coli*,* Klebsiella pneumoniae*,* Enterococcus faecalis*, and* Candida albicans* was observed at concentrations from 5 to 20 *μ*L/mL of the essential oil [10]. Iranian thyme essential oil had 54.14% of thymol and it inhibited* Candida albicans* at 0.1–20 *μ*L/mL of the essential oil [23]. Other researches have detected effective MIC to be 4 *μ*g/mL of essential oil containing 47.9% of thymol [24] and 2.4 mg/mL of essential oil containing 47.2% of thymol [25]. Essential oil containing 70.3% of carvacrol showed 0.32 *μ*g/mL MIC against* Candida albicans *[26]. Antimicrobial activity of this study was determined for 0.05–0.30% of essential oil containing oleogel, while concentration of thymol was 9.6 mg/mL and that of carvacrol was 0.07 mg/mL. Thyme essential oil is convenient to insert in oil medium; therefore a lipophilic gel was formulated. Technology process of oleogels is simple; system does not contain water; its physical, microbiological, and chemical stability is much more as compared to conventional topical bases; the lipids of oleogels support skin hydration by reducing transepidermal water loss [21]. Despite the convenience of technology, the amount of thymol decreased by 30% and it can be related to the compound volatility or other investigation aspects in future. 0.1% concentration of thyme essential oil was chosen as active substance regarding the above-mentioned aspects.

For the modeling of oleogel, the colloidal silica performed gelators role. The liquid paraffin and olive oil were mixed to compose mineral and herbal oil mixture for the oleogel formulation. The response surface central composite design was applied in order to formulate optimal composition of preparation and to investigate the relationship between ingredients and texture analysis. The influence of concentration of colloidal silica on textural parameters including firmness, cohesiveness, and consistency was found to be significant (*p* < 0.05), except on index of viscosity (*p* > 0.05). Previous studies of other researches presented high correlation between instrumental analysis and sensory evaluation of texture attributes of various skin care products [27, 28]. Oleogels have a lot of valuable properties for skin care, but texture characteristics should be acceptable for the consumers as well. Oleogel with thyme essential oil can be recommended as a skin care product with general skin protection purposes and as a natural antiseptic with pharmaceutical application.

## 5. Conclusion

GC-FID quantitative analysis of thyme essential oil showed decrease of thymol and carvacrol concentrations; therefore innovative technological step such as incorporation of volatile oil into microcapsules should be applied. Oleogel containing thyme essential oil demonstrated antibacterial activity against* Candida albicans*. Experimental design was used to optimize mixture of colloidal silica, liquid paraffin, and olive oils for the final formulation according to the evaluation of texture parameters: cohesiveness, consistency, firmness, and index of viscosity. Correlation analysis showed significant influence of colloidal silica on texture parameters. The researches about preparations with investigated thyme essential oil in the prevention and treatment of skin damage may be extended.

## Figures and Tables

**Table 1:** The set of mixtures designed by response surface model.

Number	Essential oil of thyme, %	Colloidal silica, %	Paraffin oil, %	Olive oil, % (till 100%)
(1)	0,1	4.28	30	65.62
(2)	0,1	5.00	10	84.90
(3)	0,1	5.00	50	44.90
(4)	0,1	6.75	1.72	91.43
(5)	0,1	6.75	58.28	34.87
(6)	0,1	6.75	30	63.15
(7)	0,1	6.75	30	63.15
(8)	0,1	6.75	30	63.15
(9)	0,1	6.75	30	63.15
(10)	0,1	6.75	30	63.15
(11)	0,1	8.5	10	81.40
(12)	0,1	8.5	50	41.40
(13)	0,1	9.22	30	60.68

**Table 2:** Predicted and determined texture parameters of optimal composition oleogel.

	Firmness, g	Consistency, g	Cohesiveness, g	Index of viscosity, g
Predicted	877.08	1218.8	895.33	72.35
Determined	881.08 ± 31.11	942.01 ± 40.12	723.39 ± 36.15	83.19 ± 3.16

**Table 3:** Determination of minimum inhibitory concentration (MIC) of the oleogels containing different concentrations of thyme essential oil.

Essential oil of thyme concentration in oleogels, %	Effectiveness against *Candida albicans*
0.01	—
0.03	—
0.05	Inhibited
0.10	Inhibited
0.15	Inhibited
0.20	Inhibited
0.30	Inhibited
